# Inequalities in energy-balance related behaviours and family environmental determinants in European children: changes and sustainability within the EPHE evaluation study

**DOI:** 10.1186/s12939-016-0438-1

**Published:** 2016-09-29

**Authors:** Krystallia Mantziki, Carry M. Renders, Achilleas Vassilopoulos, Gabriella Radulian, Jean-Michel Borys, Hugues du Plessis, Maria João Gregório, Pedro Graça, Stefaan de Henauw, Svetoslav Handjiev, Tommy L. S. Visscher, Jacob C. Seidell

**Affiliations:** 1 Department of Health Sciences, VU University Amsterdam, De Boelelaan 1085, 1081HV Amsterdam, The Netherlands; 2 Department of Agricultural Economics and Rural Development, Agricultural University of Athens, Athens, Greece; 3 “Carol Davila” University of Medicine and Pharmacy, Bucharest, Romania; 4 EPODE European Network Coordinating Team, Proteines, Paris, France; 5 Faculty of Nutrition and Food Sciences, University of Porto, Porto, Portugal; 6 Directorate General of Health, Lisbon, Portugal; 7 Department of Public Health, Ghent University, Ghent, Belgium; 8 Bulgarian Association for the study of Obesity and related diseases, Sofia, Bulgaria; 9 Research Centre for the Prevention of Overweight, Windesheim University of Applied Sciences Zwolle, Zwolle, The Netherlands; 10 Research Centre for the Prevention of Overweight, VU University, Zwolle, The Netherlands

**Keywords:** Health inequalities, Lifestyle behaviours, Parenting practices, EPODE, Evaluation

## Abstract

**Background:**

Increasing social inequalities in health across Europe are widening the gap between low and high socio-economic groups, notably in the prevalence of obesity. Public health interventions may result in differential effects across population groups. Therefore, the EPHE (EPODE for the Promotion of Health Equity) project analysed the added value of community-based programmes, based on the EPODE (Ensemble Prévenons l’Obésité Des Enfants-Together Let’s Prevent Obesity) model, to reduce socio-economic inequalities in energy balance-related behaviours of children and their family-environmental related determinants in seven European communities. This study presents the changes between baseline and follow-up after the one-year interventions and their sustainability one year after.

**Methods:**

This is a prospective study with a one school-year intervention, followed by one year of follow-up. In all, 1266 children (age 6-8 years) and their families from different socio-economic backgrounds were recruited at baseline. For 1062 children, information was available after one year (T_1_) and for 921 children after two years (T_2_). A self-reported questionnaire was completed by the parents to examine the children’s energy balance-related behaviours and family- environmental determinants. Socio-economic status was defined by the educational level of the mother. The Wilcoxon signed-rank test for paired data was used to test the differences between baseline and intermediate, and between intermediate and final, measurements for each of the socio-economic status groups.

**Results:**

Post-intervention effects in energy-balance related behaviours showed the following improvements among the low socio-economic status groups: increased fruit consumption (Netherlands), decreased fruit juices amount consumed (Romania) and decreased TV time on weekdays (Belgium). Whereas in only the latter case the behavioural change was accompanied with an improvement in a family-environmental determinant (monitoring the time the child watches TV), other improvements in parental rules and practices related to soft drinks/fruit juices and TV exposure were observed. A few of those effects were sustainable, notably in the case of Belgium.

**Conclusions:**

Inequalities in obesity-related behaviours could be potentially reduced when implementing community-based interventions, tailored to inequality gaps and using the EPODE methodology. Within-group changes varied widely, whereas monitoring of interventions and process evaluation are crucial to understand the observed results.

**Electronic supplementary material:**

The online version of this article (doi:10.1186/s12939-016-0438-1) contains supplementary material, which is available to authorized users.

## Background

Tackling inequalities in overweight, obesity and related determinants is high on the political and public health agenda in many European countries [1–6]. Socio-economic inequalities in obesity cases may develop in early childhood and last throughout the later stages of life [7, 8], while childhood is a critical period for shaping future behaviours. Therefore targeting children and their parents to reduce these socioeconomic inequalities is of major importance. However, most studies assess the effects of interventions in reducing overall obesity levels instead of reducing obesity-related inequalities [9]. Consequently, studies reporting the types of interventions that are effective in reducing such inequalities -particularly in children- are scarce [3, 5, 6, 9, 10].

Public health interventions may particularly reach people with a relatively high income and education and they thereby may increase inequalities, despite being effective on the general population [9, 11–15]. This is defined as the ‘intervention-generated inequality’, which evolves from the ‘inverse care law’ [16], meaning that the groups/populations mostly in need of health care are the least likely to benefit from it [12, 15, 17]. It is possible that intervention-generated inequality may happen at several (if not at any) points of the planning and the implementation of an intervention (i.e., intervention efficacy, service provision or access, uptake, compliance) [6, 12, 14, 17]. Victora et al. demonstrated that the widening of the inequality gap by the newly introduced interventions occurs due to preferential uptake of the intervention by the most advantaged groups, before the narrowing of the inequality can take place [6, 18]. In the literature, several attempts have been made to explain this phenomenon by relating it to low compliance [14], the sources of being disadvantaged [6, 18] and low participation rates [13]. Nevertheless, further research is needed to determine the specific components of interventions that result in intervention-generated inequalities [6, 17].

Several authors have attempted to specify which interventions may decrease or widen inequalities with regards to obesity. Existing evidence from universal interventions aiming at childhood obesity prevention is mixed. Bambra et al. systematically assessed the effectiveness of interventions to reduce inequalities in childhood obesity and concluded that school-based universal interventions, combining nutrition and physical activity knowledge activities had the potential to have a positive impact on low socioeconomic status children, if the interventions lasted for more than six months [19]. Other studies identified that community and/or school-based interventions were successful in reducing inequalities in obesity outcomes or did not increase them [12, 13, 15], especially when environmental change components were included [20]. Toybox, a kindergarten-based intervention aiming to increase physical activity- was only effective in the high socioeconomic kindergartens [21], whereas the “Health in Adolescents” study was effective in the middle and high education groups [11].

Another body of evidence suggests that interventions targeting the more/most disadvantaged are likely to reach the low socioeconomic groups and reduce inequalities, as long as they are strategically designed and implemented [17, 22, 23]. According to Laws et al., targeted interventions demonstrated improvement in obesity-related outcomes in low socioeconomic status populations, although most of the reviewed research was of low quality [22]. The most recent reviews suggest that upstream, community-based and multilevel interventions are more likely to reduce inequalities in health, taking into account the involvement of the hard-to-reach target groups, integrating their needs and wishes in the implementation strategies and delivering multiple interventions [12–14, 19, 22].

**Fig. 1:**
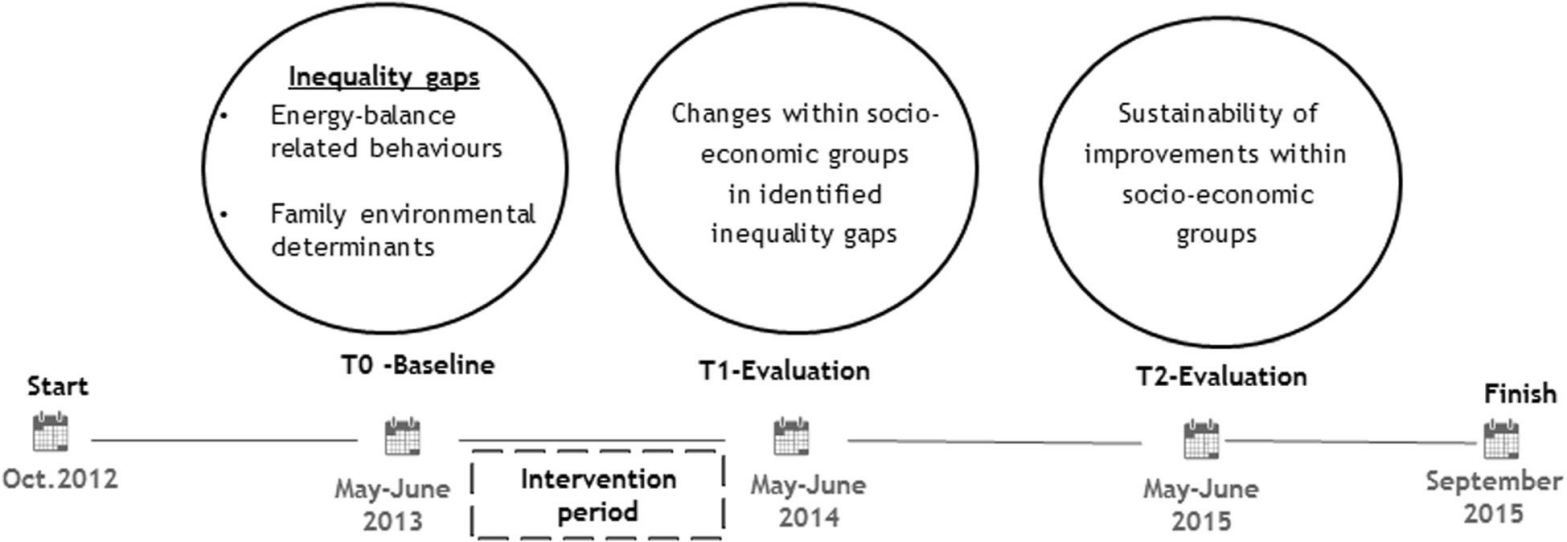
Timeline and objectives of the EPHE evaluation study

In response to that evidence and based on the reduction of health inequality in child obesity and overweight through the EPODE (Ensemble Prévenons l’Obésité Des Enfants-Together let’s prevent obesity) methodology [24–26], the EPHE (Epode for the Promotion of Health Equity) project was launched (http://www.ephestory.eu/). The overall aim of the EPHE project was to assess the impact and sustainability of EPODE to diminish inequalities in childhood obesity and overweight (Table 1). Based on scientific evidence [27–30], the EPHE scientific advisory board selected four behaviours related to obesity and overweight, which were addressed by the EPHE interventions: promotion of 1. Fruit and vegetable intake, 2. Tap water intake, 3. Active lifestyle and 4. Adequate sleep duration. The methods and framework of the EPHE project are summarised in Table 2 and the timeline is illustrated in Fig. 1.

**Table 1:** Objectives of the EPHE project

The EPHE project aims to analyse from 2012 to 2015:
▪ The added value of the implementation of an adopted EPODE methodology for the reduction of socioeconomic inequalities in health implemented by 7 European community-based programmes, focusing on four energy balance-related behaviours (fruit and vegetable consumption, tap water intake, sedentary behaviour, sleep duration) and their family-environmental determinants.
▪ Opportunities to sustain the implementation of EPHE best practices in other EU regions and member states via EU structural funds, focusing on the replicability and transferability, at a longer scale, of those to leverage the experience to develop action plans by member states and to make use of structural funds for the promotion of health equity [33].
EPHE worked at the community level in key settings to develop integrated action locally [33].

**Table 2:** Summary of the EPHE methods and framework

▪ Seven European community-based programmes, following the EPODE or similar methodology, participated in the EPHE project.
▪ The programmes recruited (at baseline) families with children aged between 6 to 9 years old from different socio-economic backgrounds, through schools.
▪ The programmes developed interventions for the whole population, each addressing the relevant inequality gaps identified at baseline [31].
▪ Intervention target: to improve energy balance-related behaviours and their family-environmental determinants of low socio-economic status families with children 6-9 years old
▪ Evaluation of the interventions’ effects after the intervention period and sustainability assessment a year after [33].

The EPHE programmes developed community-based interventions (September 2013-May 2014) addressing the four behaviours and related determinants which were unhealthier in the low socio-economic groups than in the high socio-economic groups [31]. Therefore, the objectives of the current paper are: a) to assess changes in energy-balance related behaviours and family-environmental determinants within both the high and the low education groups by comparing the baseline (T_0_) with the intermediate (T_1_) measurements, after the termination of the interventions, after one year; b) to assess the sustainability of potential improvements identified after the interventions (T_1_) a year after (T_2_). The article focuses on changes in behaviours and determinants related to the inequality gaps that were identified at the baseline measurement [33].

## Methods

The EPHE evaluation study is based on one school-year of lifestyle interventions aimed at children and their parents, followed by one year of follow-up. The interventions were carried out in seven European countries. This study aims: a) to identify differences in energy balance-related behaviours and related family-environmental determinants, between high and low status socio-economic groups, b) to assess the potential decrease of inequality gaps after tailored interventions and c) to assess the sustainability of potential improvements a year after the termination of the interventions. More information about the identified health inequalities within the EPHE study can be found elsewhere [31].

### Sample and recruitment

Seven community-based programmes, which are part of the Epode International Network and implement the EPODE methodology, participate in the EPHE project: VIASANO (Belgium), EPODE (France), PAIDEIATROFI (Greece), Maia Healthy Menu (Portugal), SETS (Romania), JOGG (The Netherlands) HEALTHY KIDS (Bulgaria); the latter programme is part of the Nestlé’s Healthy Kids programme and implements a similar methodology to EPODE. Every programme participated in EPHE project through communities within an EPODE city. We aimed at recruiting a minimum of 150 families with children aged between 6 to 8 years old in every selected EPODE community with a similar variation regarding age and ethnicity per site. We obtained convenience samples which are not necessarily representative to the country, which was beyond the scope of this study. Each of the programmes conducted the recruitment through schools. The survey obtained a permission waiver from the Medical Ethics Committee of the VU University Medical Centre. In addition, permission to research in schools was acquired from the local community and/or school authorities, where necessary. More information about sampling and recruitment are described elsewhere [32].

### EPHE intereventions

The EPHE programmes developed and implemented general community-based interventions for the selected behaviours towards the whole community, but primarily of children and parents, between September-December 2013. After the dissemination of the baseline results (September 2013), the programmes were instructed to conduct interventions tailored to the inequality gaps identified at baseline [31]. The EPHE Operational Board, comprising the national programme coordinators of each of the participant programmes, was responsible for the continuous training, empowerment and support of the local project managers of the communities, to design and implement the activities in accordance to the EPODE methodology. Thus, the board held frequent meetings and contacts to facilitate competence building and methodology transfer to the local level. Consequently, and as being the core of the EPODE methodology, various community stakeholders were involved, such as municipal representatives, school personnel, health organisations *et cetera*. This active involvement of community actors was crucial for implementing activities tailored to the community situation. To avoid stigmatization, all children of the communities (or schools in the case of the JOGG programme, municipality of Zwolle) were invited to participate to the activities, although these were tailored in behaviours and family environmental determinants, which were unhealthier in the low than in the high socio-economic groups. However, due to time constrains the majority of the programmes were able to target only the energy-balance related behaviours and not the determinants. Examples of activities held within the EPHE project are games, workshops and educational materials on healthy diet, psychical activity and sleep. More information about the type of implemented activities, stakeholder involvement and implementation methods are included elsewhere [33].

### Data collection

School teachers distributed the questionnaires, including an informed consent form, to the children who consequently delivered them to their parents, after the intervention period between May/June 2014 (T_1_) and a year later, May/June 2015 (T_2_). After a specified period of one to two weeks, the completed questionnaires were returned likewise to the teachers. Thereafter, the EPHE project managers collected the questionnaires from the schools and only the ones including a signed informed consent form were taken into consideration. In order to ensure the confidentiality of the data, a process to guarantee anonymity of participant families was applied [33].

### EPHE parental questionnaire

It is well documented that a sustained positive energy balance in children is associated with several lifestyle behaviours, such as, low consumption of fruit and vegetables, high sugar intake, high fat intake, unhealthy snacking, physical inactivity, high screen time and short sleep duration [27–30]. In addition studies have demonstrated associations between the family environment parental practices, rules and behaviours and the children’s energy-balance related behaviours [34–36]. The EPHE scientific advisory board selected to address the following behaviours: fruit and vegetable intake, tap water intake, sugary beverages intake (i.e., fruit juices and soft drinks), screen exposure (i.e., television and computer) and adequate sleep duration. Furthermore, associated family-environmental determinants were assessed [34–36].

In order to assess differences in energy-balance related behaviours and their determinants among different socio-economic groups (inequality gaps), a self-administered parental questionnaire was developed. The EPHE parental questionnaire was developed using items from relevant, validated questionnaires addressed in European populations: ENERGY parent and child questionnaires [34], the Pro-children child questionnaire [35] and its updated version PRO-GREENS [36], European Health Examination Survey questionnaire [37], European Social Survey questionnaire [38], United States Department of Agriculture questionnaire [39]. Additional items were constructed in the cases where, to our knowledge, no validated items or questionnaires existed.

### Assessment of energy-balance related behaviours

The questionnaire assessed four energy-balance related behaviours of the child: 1. fruit and vegetable consumption; 2. soft drink/fruit juices and water consumption; 3. TV or computer screen time and 4. sleep duration, as well as determinants related to the social and physical environment of the child, within the family setting. In order to keep the length of the questionnaire within acceptable limits, we had to prioritise the many aspects of behaviour that could be relevant. The EPHE scientific advisory board decided (in consultation with experts) to keep sedentary behaviour as the indicator of physical activity. Other relevant aspects, which were not included, were snacks and meals (such as breakfast, lunch and dinner).

The consumption of fruits and vegetables was assessed by food frequency questions, referring to a usual week and measured on an 8-point Likert scale (1. Never - 8. Every day, more than twice a day) [32, 35, 36]. The consumption of fruit juices, soft drinks and diet soft drinks was measured by means of weekly frequency and amount consumed. The frequency was measured on a 7-point Likert scale (1. Never - 7. Every day, more than once a day) [32, 34]. The amount was measured by two items for fruit juices and three items for soft and diet soft drinks, assessing how many glasses (or small bottles; 250 ml), cans (330 ml) or big bottles (500 ml) the children drink [32, 34]. The amount was calculated by summing up the portions. In order to measure water consumption, two questions were constructed to measure the daily frequency (1. Never - 7. More than six times a day) and number of glasses consumed when drinking water (1. None - 6. five or more glasses). Sedentary behaviour is assessed by means of daily time spent in television (TV) viewing and time of computer (PC) use, for the week and the weekend days separately, measured on a 9-point Likert scale (1. Not at all - 9. 4.0 or more hours a day) [32, 34]. The total screen time was calculated by the sum of weekly (hours per weekday*5 + hours per weekend day*2) TV and PC use. Furthermore, two questions informed by the ENERGY parent questionnaire assess the sleeping habits of the child (1. Sleeping routine; 2. Sleep duration per week/weekend-day) [32, 34].

### Assessment of determinants

The determinants assessed refer to the social and physical family environment of the child. These were mainly assessed by one item and most of them were measured on a 5-point Likert-type scale (0. Never - 4. Always or -2. Fully disagree - 2. Fully agree), unless otherwise stated below and in the tables of this article; more details are described in Mantziki et al. [32]. The social environmental determinants are: a) for *fruit and vegetable* consumption, i. Parental demand (0. Never - 4. Yes, always), ii. Parental allowance (0. Never - 4. Yes, always), iii. Active encouragement (-2. Fully disagree - 2. Fully agree) and iv. Facilitating (0. Never - 4. Yes, always) and v. Parental knowledge on recommendations (1. no fruit – 8. 5 pieces per day [32, 35, 36]; b) for fruit juice/soft drink consumption and TV viewing/computer exposure, i. Paying attention/monitoring (0. Never - 4. Always), ii. Parental allowance (0. Never - 4. Always), iii. Negotiating (0. Never - 4. Always), iv. Communicating health beliefs (0. never - 4. always), v. Avoid negative modelling (0. never - 4. always), vi. Parental self-efficacy to manage child’s intake (0. never - 4. always), vii. Rewarding/comforting practice (0. Never - 4. Always), viii. Conducting energy-balance related behaviour together with the child (1. Never- 8. Every day more than once; for TV viewing/computer time the scale is ‘0. Never - 4. Always’) [32, 34]. The physical environmental determinants are: a) for the consumption of *fruit and vegetables*, i. home availability (0. Never – 4. Always) and ii. Situation specific habit (-2. Fully disagree - 2. Fully agree) [32, 35, 36] b) for *fruit juices/soft drinks* consumption, i. Home availability (0. Never - 4. Yes, always) and ii. Situation specific habit (1. Yes - 2. No) [32, 34]; and c) for *TV viewing\computer* exposure, i. Availability (1. Yes - 2. No) ii. Situation specific habit (TV on during mealtime) (1. Every day – 5. Never) [32, 34].

### Socioeconomic measures

The socio-economic status indicators measured were parental employment status, perception of income position, parental educational level, parental sector of employment. The aforementioned variables are described in detail by Mantziki et al. [32]. Knowing that maternal educational level has been classified as a good social factor explaining differences in nutritional outcomes in children [40–42], for the current study, the samples were divided into two groups based on the *educational level of the mother* (low-high). The educational level was assessed by a 6-point ordinal scale, measuring the years of education accomplished (1. Less than 6 years -6. More than 17 years; Table 3). For each country’s sample the median of the educational level was used as the cut-off point to define the educational level of the mother (low-high).

**Table 3:** Socio-demographic characteristics of the EPHE population per country after the interventions and (T_1_) and after one year (T_2_)

Country	Gender		Age child (years)	Age of mother^b^		Educational level mother		Total n^a^
	Boys (%)	Girls (%)	Mean (SD)	<30 (%)	>30 (%)	High (%)	Low (%)	
T_1_								
Belgium	82 (51,6)	77 (48,4)	7,62 (,52)	18 (14,0)	113 (86,0)	73 (45,6)	87 (54,4)	160
Bulgaria	71 (46,1)	83 (53,9)	8,68 (,50)	5 (4,0)	122 (96,0)	116 (75,3)	38 (24,7)	154
France	37 (35,2)	68 (64,8)	6,87 (,73)	22 (22,6)	55 (77,4)	41 (39,0)	64 (61,0)	105
Greece	67 (51,1)	64 (48,9)	8,32 (,63)	2 (1,8)	108 (98,2)	70 (53,4)	61 (46,6)	131
Portugal	90 (48,9)	94 (51,1)	7,87 (,76)	12 (7,3)	152 (92,7)	86 (46,7)	98 (53,5)	184
Romania	89 (55,6)	71 (44,4)	8,31 (,49)	21 (15,9)	111 (84,1)	86 (53,1)	76 (46,9)	162
The Netherlands	28 (43,1)	37 (56,9)	8,51 (,65)	1 (1,9)	53 (98,9)	46 (70,8)	19 (29,2)	65
Total	468 (48,0)	498 (52,0)	8,02 (,82)	81 (9,9)	732 (90,1)	516 (53,8)	444 (46,2)	961
T_2_								
Belgium	69 (50,0)	69 (50,0)	8,5 (,50)	12 (10,0)	108 (90,0)	74 (53,2)	65 (48,6)	139
Bulgaria	60 (45,5)	72 (54,5)	8,98 (,12)	9 (7,5)	112 (92,5)	97 (72,4)	37 (27,6)	134
France	29 (35,8)	52 (64,2)	7,87 (,68)	12 (15,6)	65 (84,4)	32 (39,5)	49 (60,5)	81
Greece	53 (50,0)	53 (50,0)	8,88 (,33)	0 (0,0)	95 (100)	50 (46,7)	57 (53,3)	107
Portugal	73 (47,7)	80 (52,3)	8,64 (,48)	9 (6,3)	134 (93,7)	82 (53,6)	71 (46,4)	153
Romania	78 (54,2)	65 (45,1)	8,94 (,23)	6 (4,4)	129 (95,6)	77 (53,5)	67 (46,5)	144
The Netherlands	16 (44,4)	20 (55,6)	8,89 (,32)	0 (0,0)	36 (100)	27 (75,0)	9 (25,0)	36
Total	378 (47,9)	411 (52,1)	8,70 (,52)	6,9	93,1	53,7	46,3	794

^a^Total number of subjects that were followed-up and provided information for the educational level of the mother’; the number reflects the subjects included in the analysis

^b^The analysis includes the age of the mother only when the mother was the respondent; the age of the second parent was not assessed; Response categories: 1 = Below 20, 2 = 21-24, 3 = 25-30, 4 = 31-35, 5 = 36-40, 7 = Above 40. Number of subjects included in “age of mother” per country were a. at T_1_ :Belgium = 129, Bulgaria = 127, France = 97, Greece = 110, Portugal = 164, Romania = 132, The Netherlands = 54, Total = 813; b. at T_2_ : Belgium = 116, Bulgaria = 121, France = 73, Greece = 86, Portugal = 136, Romania = 120, The Netherlands = 54, Total = 684

### Statistical analysis

The Wilcoxon signed-rank test for the ordinal and McNemar’s test of paired proportions for the binomial variables were used to detect differences in energy-balance related behaviours and determinants a. between T_0_ and T_1_ within the low and within the high education groups, for the variable where an inequality gap was identified at T_0_; b. between T_1_ and T_2_ within both the low and high education groups, in the variables where an improvement was observed between T_0_-T_1_. The complete follow-up samples for were analysed, which differed in number between T_1_ and T_2_. Here we present medians and quartile ranges for the ordinal variables and percentages for the binomial variables, in order to illustrate the differences within both the low and high education groups. Knowing that the mean ranks produced by non-parametric tests are not always sufficiently informative and that differences in spread may be equally important as differences in medians [43], further assessment of frequencies and distributions was explored. The results of the additional assessments are not presented in this article due the large amount of information.

All analyses were conducted using the SPSS software v. 21.0 package (IBM Corp., Armonk, NY, USA).

Adjustment for multiple testing was conducted for the intermediate measurements (T_1_), using the Benjamini and Hochberg method [44], using the Stata software v. 13 package (StataCorp. 2013. *Stata Statistical Software: Release 13*. College Station, TX: StataCorp LP).

## Results

**Fig. 2:**
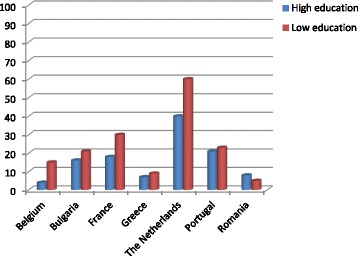
Percentage of population lost-to follow-up at T_1_ per educational group per country

**Fig. 3:**
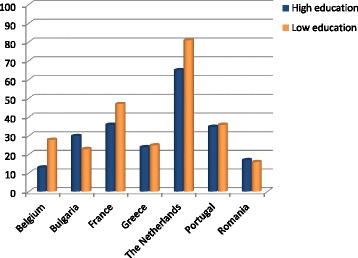
Percentage of population lost-to follow-up at T_2_ per educational group per country

A total of 1061 children and their families were involved in the survey at the end of the interventions (T_1_) and 921 in the final survey one year after the end of the interventions (T_2_). Due to missing data in the variable ‘educational level of mother’, finally 961 and 794 subjects were included in the analysis in T_1_ and T_2_ respectively. On average, the percentage of those cases lost to follow-up at T_1_ was 30 %, whereas it increased to 34 % at T_2_. The dropout of the low education group was higher in nearly all countries in both follow-up periods, as illustrated in Figs. 2 and 3.

Tables 4, 5, 6, 7 present only the changes in behaviours that differed between children from low and high socio-economic background (inequality gaps) at baseline [31]. Similarly the respective changes in determinants are presented in Additional files 1, 2, 3, 4 and 5. Given the large amount of data, we chose to discuss the statistically significant changes only. In addition, considering the second objective of the study- to assess the sustainability of the improvements that occurred between pre and post-intervention period, Table 8 and Additional file 6 illustrate the sustainability of such changes.

**Table 4:** Within-group comparison of median values and quartiles (q_1_-q_3_) between T_0_-T_1_ for weekly dietary intake per education group

	T_0_		T_1_	
Education level	High	Low	High	Low
Country				
Fruit consumption (frequency/week) ^a^				
Portugal	7 (6–7)	6 (5–7)	7 (6–7)	6 (5–7)
Romania	6 (4–6)	5 (4–6)	6 (4–6)	6 (4–6)
The Netherlands	6 (6–7)	5 (4–6)**	6 (6–7)	6 (5–7)**
Salad/grated vegetables consumption (frequency/week) ^a^				
Portugal	6 (4–7)	5 (4–7)	6 (5–7)	6 (4–6)
Cooked vegetables consumption (frequency/week) ^a^				
Portugal	7 (6–7)	6 (5–7)	7 (6–7)	6 (5–7)
Romania	5 (4–6)	4 (3–6)	5 (4–6)	4 (4–6)

Comparison between the educational groups of each country and the total sample with Wilcoxon signed rank test. Rounded values are presented

^a^Response categories: 1.Never 2.Less than one day per week 3.One day per week 4.2-4 days a week 5.5-6 days a week 6.Every day, once a day 7.Every day, twice a day 8.Every day, more than twice a day

**significant within-group difference at .01

**Table 5:** Within-group comparison of median values and quartiles (q_1_-q_3_) T_0_-T_1_ for weekly beverage intake per education group

	T_0_		T_1_	
Education level	High	Low	High	Low
Country				
Fruit juices frequency^a^				
Romania	4 (3–6)	4 (2–4)	4 (3–4)	3 (3–4)
Fruit juices amount (ml)^b^				
Belgium	500 (250–580)	580 (500–750)	500 (250–580)	580 (250–830)
Bulgaria	580 (500–830)	830 (580–1160)	580 (540–830)	580 (250–830)
Romania	580 (250–830)	580 (580–1060)**	580 (250–830)	580 (580–580)**
The Netherlands	250 (250–500)	250 (250–580)	500 (250–580)	500 (250–580)
Soft drinks frequency^a^				
Portugal	2 (1–3)	3 (2–3)	2 (1–3)	2 (2–3)
Romania	2 (1–3)	3 (2–4)	2 (1–3)	3 (2–4)
Soft drinks amount (ml)^b^				
Portugal	250 (125–580)	580 (250–580)	250 (250–580)	580 (250–580)
Romania	580 (125–915)	1000 (500–1160)	580 (250–1080)	580 (250–1080)

Comparison between the educational groups of each country and the total sample with Wilcoxon signed rank test. Rounded values are presented

^a^Response categories: 1.Never 2.Less than once a week 3.Once a week 4.2-4 days a week 5.5-6 days a week 6.Every day, once a day 7.Every day, more than once a day

^b^The indicated amounts are derived from the sum of the respective question items; J3a and J3b and K3a, K3b and K3c for fruit juices amount and soft drinks amount respectively [31]. The variables are categorical with specific values of ml in each category

**significant within-group difference at .01

**Table 6:** Within-group comparison of median values and quartiles (q_1_-q_3_) T_0_-T_1_ for screen exposure per education group

	T_0_		T_1_	
Education level	High	Low	High	Low
Country				
TV weekdays (h/day) ^a^				
Belgium^d^	3 (2–4)	5 (3–6)**	3 (3–4)	4 (3–5)**
France	3 (2–4)	4 (3–4)	4 (1–4)	4 (3–5)
Greece	3 (2–4)	4 (3–4)	3 (2–4)	3 (3–4)
Portugal^c^	3 (2–4)	3 (3–4)	3 (3–4)	3 (3–4)
Romania^c^	3 (3–4)	4 (3–6)	3 (3–5)	4 (3–6)
TV weekend days (h/day) ^a^				
Belgium^d^	5 (4–7)	7 (5–8)	5 (4–7)	6 (4–7)
France	5 (4–7)	6 (4–8)	4 (4–6)	5 (4–7)
Portugal	5 (4–6)	6 (4–7)	5 (4–6)	6 (4–7)
PC weekdays (h/day) ^a^				
Belgium^d^	1 (1–2)	2 (1–3)	1 (1–2)	2 (1–3)
Bulgaria	2 (2–3)**	3 (2–3)	3 (2–3)**	3 (2–3)
PC weekend days (h/day) ^a^				
Bulgaria	3 (2–4)*	4 (3–5)	4 (3–5)*	4 (3–4)
Romania^c^	4 (2–5)	3 (1–5)***	4 (3–6)	5 (3–6)***
Total screen time (h/week) ^b^				
Belgium^d^	12.5 (9–19)	19.5 (12–25)	12 (9–18)	17 (11–22.8)
Bulgaria	18 (12–26)**	23.50 (13.5–30)	20.5 (13.5–29)**	24 (16–30)
France	14 (9–24)	17.5 (11–22.5)	10 (16–22)	18.3 (11.4–23)
Greece	13.5 (9.5–20.5)	18 (13–22.5)	13.5 (9.5–20)	18.5 (13.5–26)
Portugal^c^	14.5 (10–20)	17 (11–23)	15 (12–22)	17 (12.5–22.5)

Comparison between the educational groups of each country and the total sample with Wilcoxon signed rank test. Rounded values are presented

^a^Response categories: 1.Not at all 2.30 min/day 3.1 h/day 4.2 h/day 5.2,5 h/day 6.3 h/day 7.3,5 h/day 8.4 or more h/day

^b^The indicated amounts of hours are derived from the sum of the respective question items for TV (T1a and T1b) and PC time (T4a and T4b) [31]. The variables are categorical with specific values of hours in each category

^c^: the variables PC time for weekdays and weekend-days are measured with an extra response category for 1,5 h/day (coded as 4); as such the items include 9 response categories. This does not apply for the results of the total sample

^d^: the variables TV/PC time for weekdays and weekend-days are measured with an extra response category for 1,5 h/day (coded as 4); as such the items include 9 response categories. This does not apply for the results of the total sample

**, ***: significant within-group difference at .01 and .001 respectively

**Table 7:** Within-group comparison of median values and quartiles (q_1_-q_3_) T_0_-T_1_ for sleep hours per educational group

	T_0_		T_1_	
Education level	High	Low	High	Low
Country				
Sleep duration weekdays (h/day) ^a^				
Portugal	3 (2–3)	2 (2–3)	3 (2–3)	2 (2–3)
The Netherlands	3 (3–3)	3 (2–3)	3 (3–3)	3 (3–3)
Sleep duration weekend days (h/day) ^a^				
The Netherlands	3 (3–3)	3 (2–3)	3 (3–3)	3 (3–3)

^a^ Response categories: 1. 6 h or less/per night 2.7 h/per night 3.8 h/per night 4.9 h/ per night 5.10 h/per night 6.More than 10 h per night

**Table 8:** Within-group comparison of median values and quartiles (q_1_-q_3_) between T_1_-T_2_ for energy-balance related behaviours per education group

	T_1_		T_2_	
Education level	High	Low	High	Low
Country				
Fruit consumption (frequency/week) ^a^				
The Netherlands	7 (6–7)	6 (5–7)	7 (6–7)	6 (4–6)
Fruit juices amount (ml)^b^				
Romania	580 (250–830)	580 (250–580)***	580 (580–830)	580 (580–830)**
TV time weekdays (h/day) ^c^				
Belgium^d^	3 (2–4)	4 (3–5)	3 (2–4)	4 (3–5)

Comparison between the educational groups of each country and the total sample with Wilcoxon signed rank test. Rounded values are presented

^a^Response categories: 1. Never 2. Less than one day per week 3. One day per week 4. 2-4 days a week 5. 5-6 days a week 6. Every day, once a day 7. Every day, twice a day 8.Every day, more than twice a day

^b^The indicated amounts are derived from the sum of the respective question items; J3a and J3b and K3a, K3b and K3c for fruit juices amount and soft drinks amount respectively [31]. The variables are categorical with specific values of ml in each category

^c^Response categories: 1.Not at all 2.30 min/day 3.1 h/day 4.2 h/day 5.2,5 h/day 6.3 h/day 7.3,5 h/day 8.4 or more h/day

^d^:the variables TV/PC time for weekdays and weekend-days are measured with an extra response category for 1,5 h/day (coded as 4); as such the items include 9 response categories

**, ***: significant within-group difference at .01 and .001 respectively

### Changes in energy balance-related behaviours and their sustainability

Tables 4, 5, 6, 7 shows changes in dietary intake, beverage intake, screen exposure and sleep hours, respectively, between the pre- and post- intervention period. Some behaviours were improved among the low socio-economic groups, reducing the inequality gaps between children from low and high socio-economic background that were identified at baseline. However, a few worsening trends were observed as well within both the low and the high educational groups at T_1_; besides that, few of the improved changes were sustained at T_2_.

More specifically, the frequency of fruit intake increased significantly within the Dutch low education group (Table 4), reaching the same frequency as in the high education group. A small, but statistically significant decrease in the consumption of fruit juices was seen within the Romanian low education group (Table 5). TV time during weekdays decreased among the Belgian children from the low educational group (Table 6). Moreover, computer time both during weekdays and during weekend days increased significantly within the Bulgarian high education group, resulting in higher screen exposure during the week (Table 6). Computer time during weekends also increased in the Romanian sample, however, within the low education group (Table 6). No notable changes were found with respect to sleep hours (Table 7).

A year after the interventions, two of the aforementioned changes were sustained, namely the increased fruit intake among the Dutch low education group and the decrease of TV time spent on weekdays among the Belgian low education status group (Table 8).

### Changes in determinants of energy balance-related behaviours and their sustainability

Similarly to the behavioural changes, we found a few statistically significant changes related to inequality gaps identified at baseline in the determinants of the assessed behaviours, within the low and within the high education groups in all countries, and again few of the reduced gaps were sustained.

In particular, no noteworthy changes were observed related to the determinants of fruit and vegetable consumption (Additional file 1). Parental practices related to the consumption of fruit juices improved in families with a low educational status background in Belgium (parental allowance), Greece (negotiate parental allowance) and Portugal (rewarding/comforting practice; Additional file 2). The latter was sustained a year after the interventions (Additional file 6).

For the determinants of soft drinks consumption, the observed effects were mixed. As illustrated in Additional file 3, in France the children of highly educated mothers complained more often when soft drinks were not allowed (nagging), whereas Romanian parents from a low educational background increased the frequency of drinking soft drinks in the presence of their child (avoid negative modelling; Additional file 3) compared to baseline. In contrast, a noteworthy change in Portugal was observed, namely the decreased home availability of soft drinks among the low education group (Additional file 3), which was maintained a year after the interventions (Additional file 6).

More changes were observed in the determinants of screen exposure. Parental practices and rules improved in some countries within families from a low educational background (i.e., increased monitoring of child’s TV time (Belgium), increased efficacy to control TV exposure of the child (Greece), decreased allowance of TV watching (Portugal) (Additional file 4), except in the Netherlands (avoid less often computer use in the presence of the child) (Additional file 5). Among the high education group, parental negotiation for the allowed TV time increased in France, indicating less strict rules (Additional file 4). All of the aforementioned improvements within the low education group were being sustained a year after the interventions (Additional file 6).

### Results (T_1_) after multiple testing adjustments

Adjustments for multiple testing resulted in critical *p*-values lower than 0.05 (ranging from 0.000316 to 0.002532), as initially set by the authors (Additional file 7). Consequently, fewer of the differences found within the education groups of each of the samples (based on α = 0.05) were significant, based on the adjusted lower threshold (Additional file 7). As an illustration, the statistically significant differences within the Portuguese low education status group were initially 3 and after the adjustments this was reduced to 1 (Additional file 7). It was noteworthy that the decrease of TV time during weekdays among the Belgian low education group remained statistically significant (Additional file 7).

## Discussion

After a one school-year (8/9-months) intervention period aiming at reducing inequality gaps between low and high socio-economic status children and their families in health behaviours and determinants, an improvement of three energy-balance related behaviours among the low socio-economic status groups was observed, namely an increase of fruit consumption (Netherlands), decrease in the amount fruit juices consumed (Romania) and decrease of TV time on weekdays (Belgium). Whereas in only the latter case was the behavioural change accompanied by an improvement in a family-environmental determinant (monitoring the time the child watches TV), other improvements in parental rules and practices related to soft drinks/fruit juices and TV exposure were observed. These results, however, cannot be exclusively attributed to the EPHE interventions, given that causality is not analysed in this study.

Our results are supported by two systematic reviews, which found positive changes in intervention studies targeting behavioural changes, such as increase of physical activity and fruit and vegetable intake, decrease of screen time and intake of sugary beverages [19]. Most of these effective interventions were targeted at the low socio-economic status population, whereas only one was universal as the EPHE ones [19]. With regard to the changes we found in parental practices, observed primarily within the low socio-economic status groups, the improved values were similar or inclined towards the ones of the subjects of the respective high socio-economic status groups. These positive changes contradict the commonly observed phenomenon of the intervention-generated inequality [9, 11–15, 17]. Thus it seems that it is possible through universal interventions to reach, improve and even sustain the improvement of parental practices, including in low socio-economic status groups. This may even be related to sustained changes in behaviour, as indicated by the sustained decrease in TV time on weekdays (Belgium), which may in turn be associated with the sustained increase in monitoring the child’s time spent watching television.

Nevertheless, a few statistically significant and usually small changes were observed in the assessed outcomes between the pre- and post-intervention period within the low socio-economic status groups and even fewer were sustained one year after. Consequently, some of the inequality gaps were decreased and sustained, but not all of them. One reason for this, apparently, was the short preparation time for designing the interventions, which impeded the programmes to implement those interventions targeted at inequality gaps in the determinants, as initially intended. Another reason was probably the short duration of the interventions and consequently their low intensity to be able to result in sustainable behaviour change. Two reviews concluded that intervention studies, of moderate to high quality, improved energy-balance-related behaviours when implemented for more than six months, whereas community-based interventions delivered universally also reduced obesity-related outcomes of other kinds in all population groups in the long-term (>6 months) [9, 19]. Furthermore, a widening of inequalities was prevented through a multi-level, community capacity-building approach, in the medium to longer period (≥6 months) [9, 19]. It is worth mentioning the Fleurbaix–Laventie Ville Sante´ study, based on the EPODE methodology, which showed a reduction in obesity prevalence in the lower socio-economic status group compared to the respective control group, only after conducting 12 years of community-based interventions [26]. Furthermore, Magneé et al. concluded from their assessement of universal interventions, that socio-economic inequalities in physical activity, diet or prevention of obesity are most likely to be reduced through intensive community level interventions, underlining the importance of tailoring interventions to the needs of low socio-economic status populations [13]. Whereas we considered the tailoring as selecting behaviours and determinants of behaviours that differed and therefore should be our target, the literature shows that tailoring should involve an investigation of the target population [45–47] and require participation of the target population in the development of interventions [48]. This was not possible in the EPHE project because of time constrains.

### Strengths and limitations

To our knowledge, this is the first evaluation study that provides data on socio-economic inequalities in family-environmental determinants associated with energy-balance related behaviours across a wide variety of European countries. Translation and back translation procedures in the development of the questionnaires enabled comparisons of the study results across countries. The cross-cultural character of the sample enables the exploration of inequalities in factors that have been strongly associated with childhood obesity. Such studies may be especially important in the light of the rapidly changing economic circumstances in many parts of the Europe. In addition, our results provide new insight into energy-balance behaviours and their determinants, which should be the focus for the development of effective interventions aimed at reducing inequalities in childhood obesity.

However, our study has certain limitations. For the purpose of the EPHE evaluation study, the participant programmes were selected on the basis of towns or locations that were already actively involved with EPODE. They may not be representative of the countries in which they are located and may have resulted in the selection of towns where already ongoing community-based interventions had resulted in changes in behaviour. In addition, the schools from which the samples were recruited were selected based on accessibility and convenience criteria. The results of this study must be therefore interpreted and generalized with caution. Moreover, the higher drop-out of subjects from the low education group may have impeded the power of this study to detect significant effects after the interventions and/or their potential sustainability.

In addition the population of the middle socio-economic status group was divided among the population of high and low socio-economic status, due to the small number of subjects in the lowest educational category. Thus the ability to detect big differences among the cohorts might be limited. Another weakness of this study could be that we used the educational level of the mother as a proxy for socio-economic status, instead of using a wider set of indicators. Although the parental education level has been characterised as an adequate socio-economic indicator by relevant and more elaborative studies [40–42], this still reduces the strength of detecting absolute inequalities. It is important to mention that the power of the associations observed is decreased, due to loss-to-follow-up, especially in the Dutch sample, of which the size was considerably reduced. Furthermore this study reports selectively on the statistically significant changes, which were considerably reduced after adjustments for multiple testing. Yet, we consider our results important as they give indications that improvements in lifestyle behaviours among low socio-economic status groups are possible. Moreover it seems that small changes may contribute to tackling the public health problem of obesity on population level.

Moreover, the data were self-reported and recall bias and/or socially desirable answers are possible as in nearly all large scale community interventions. Besides, a disadvantage of using the same questionnaire across all countries was that not all items are as relevant for all countries. Furthermore, errors from the constructed items are possible, given that they were not validated. In addition, this is an effect evaluation, which did not use a control group. Thus, conclusions about causality cannot be drawn, the effects cannot be exclusively attributed to the interventions, and neither can conclusions on the quality of the interventions that were carried out be drawn.

### Implications for public health practice

The results of our study seem to support the view that improvement in energy-balance related behaviours and parental rules and practices in the low socio-economic status populations is feasible by implementing interventions designed on the basis of studied gaps, and tailored to the behaviours and determinants that differed between low and high socio-economic status families, within an existing health promotion programme that is already targeting the whole population. However the short duration leads to only moderate favourable changes, besides the very low potential to sustain improvement.

### Implications for public health research

Inequalities in family environmental determinants- such as parental rules and availability of fruit, vegetables, sugary-sweetened beverages and screens in the personal space of the child- may be addressed with more success by upstream, high intensity, long-term and multi-level interventions. Therefore further studies including the use of a control group are needed, to establish the ability of such interventions to reduce inequalities in obesity-related determinants and behaviours. Further studies could assess whether existing social-marketing strategies could help or whether such strategies should be intensified when aiming at reducing socio-economic gaps.

## Conclusions

The improvements in behaviours and determinants observed among children of both high and low socio-economic status, indicate that inequalities in obesity-related behaviours could be potentially reduced when implementing community-based interventions universally delivered, targeting those behaviours and determinants where inequalities exist, and being developed according to the EPODE methodology. The results showed large variability in the observed changes after the implemented interventions, while the monitoring of interventions and process evaluation is crucial to understand the observed results. Future research is necessary, evaluating more tailored interventions and upstream and environmental interventions that require targeted health policies.

## Additional files

Additional file 1: Within-group changes (T_0_-T_1_) in median values (q_1_-q_3_) in the determinants of fruit and vegetable consumption. (DOCX 20 kb) Additional file 2: Within-group changes (T_0_-T_1_) in median values (q_1_-q_3_) in the determinants of fruit juices consumption. (DOCX 18 kb) Additional file 3: Within-group changes (T_0_-T_1_) in median values (q_1_-q_3_) in the determinants of soft drinks consumption. (DOCX 18 kb) Additional file 4: Within-group changes (T_0_-T_1_) in median values (q_1_-q_3_) in the determinants of TV exposure. (DOCX 21 kb) Additional file 5: Within-group changes (T_0_-T_1_) in median values (q_1_-q_3_) in the determinants of PC exposure. . (DOCX 18 kb) Additional file 6: Within-group changes (T_1_ - T_2_) in median values (q_1_-q_3_) in determinants per behavior. (DOCX 20 kb) Additional file 7: Corrected critical p-values after adjustment for multiple testing (T_1_). (DOCX 34 kb)

## Abbreviations

EPODE: Ensemble Prévenons l’Obésité Des Enfants-Together let’s prevent obesity
EPHE: EPODE for the promotion of health equity
PC: Personal computer
