# Supplementary material for: Inequalities in energy-balance related behaviours and family environmental determinants in European children: changes and sustainability within the EPHE evaluation study
Source: Int J Equity Health. 2016 Sep 29;15:160. doi: 10.1186/s12939-016-0438-1 (PMC5041563; doi:10.1186/s12939-016-0438-1)
Supplement: Additional file 1: — Within-group changes (T0-T1) in median values (q1-q3) in the determinants of fruit and vegetable consumption. (DOCX 20 kb) [file 12939_2016_438_MOESM1_ESM.docx]

**Additional file 1**. Within-group changes (T_0_-T_1_) in median values (q_1_-q_3_) in the determinants of fruit and vegetable consumption.

| **Determinants**  **by country** | **Fruit consumption** | | | | | | | |  |  |
| --- | --- | --- | --- | --- | --- | --- | --- | --- | --- | --- |
|  | **T_0_** | | | | **T_1_** | | | |  |  |
| **Education group**  **Belgium** | High | | Low | | High | | Low | |  |  |
| Home availability  *Never (0) - yes, always (4)* | 3 (3-4) | | 3 (2-4) | | 4 (3-4) | | 3 (3-4) | |  |  |
| **Education group**  **Bulgaria** | High | | Low | | High | | Low | |  |  |
| Parental allowance  *Never (0 ) - yes, always (4)* | 4 (4-4) | | 4 (3-4) | | 4 (4-4) | | 4 (4-4) | |  |  |
| **Education group**  **Greece** | High | | Low | | High | | Low | |  |  |
| Habit to eat fruit/vegetables daily  *(-2) fully disagree-(2) fully agree* | 1 (0-1) | | 1 (1-2) | | 1 (1-2) | | 1 (0-2) | |  |  |
| **Education group**  **Portugal** | High | | Low | | High | | Low | |  |  |
| Performing EBRB together with the child  *Never (0 ) - yes, always (4)* | 2 (1-2) | | 2 (1-2) | | 2 (2-2) | | 2 (1-2) | |  |  |
| Home availability  *Never (0) - yes, always (4)* | 4 (3-4) | | 3 (3-4) | | 4 (3-4) | | 3 (3-4) | |  |  |
| **Education group**  **Romania** | High | | Low | | High | | Low | |  |  |
| Facilitating  *Never (0) - yes, always (4) (F31)* | 2 (2-3) | | 2 (2-3) | | 2 (2-3) | | 2 (2-3) | |  |  |
| Home availability  *Never (0) - yes, always (4)* | 4 (3-4) | | 3 (2-4) | | 4 (3-4) | | 3 (2-4) | |  |  |
| **Education group**  **The Netherlands** | T_0_ | | T_0_ | | T_1_ | | T_1_ | |  |  |
| Parental Demand  *Never (0) - yes, always (4)* | 3 (3-4) | | 3 (3-3) | | 3 (3-4) | | 3 (2-4) | |  |  |
|  | | | **Vegetable consumption** | | | | | | | |
|  |  |  | **T_0_** | | | | **T_1_** | | | |
| **Education group**  **Greece** | | | High | | Low | | High | | Low | |
| Parental knowledge on recommendations  *None (1) - 5 or more pieces/portions per day (8)* | | | 5 (4-5) | | 4 (4-5) | | 4 (4-5) | | 4 (3-5) | |
| **Education group**  **Portugal** | | | High | | Low | | High | | Low | |
| Parental allowance  *Never (0 ) - yes, always (4)* | | | 3 (3-4) | | 3 (3-4) | | 3 (3-4) | | 3 (3-4) | |
| Facilitating  *Never (0) - yes, always (4) (F31)* | | | 3 (3-4) | | 3 (2-3) | | 3 (3-4) | | 3 (2-3) | |
| Parental knowledge on recommendations  *None (1) - 5 or more pieces/portions per day (8)* | | | 5 (5-6) | | 5 (5-6) | | 5 (5-6) | | 5 (4-6) | |
| Home availability  *Never (0) - yes, always (4)* | | | 3 (3-4) | | 3 (2-4) | | 3 (3-4) | | 3 (3-4) | |
| **Education group**  **Romania** | | | High | | Low | | High | | Low | |
| Performing EBRB together with the child  *Never (0 ) - yes, always (4)* | | | 2 (1-2) | | 2 (2-2) | | 2 (2-2) | | 2 (2-2) | |
| Facilitating  *Never (0) - yes, always (4) (F31)* | | | 3 (3-4) | | 3 (2-3) | | 3 (3-3) | | 3 (2-3) | |
| **Education group**  **The Netherlands** | | | High | | Low | | High | | Low | |
| Facilitating  *Never (0) - yes, always (4) (item F31)* | | | 4 (3-4) | | 3 (3-4) | | 4 (3-4) | | 4 (3-4) | |
| Habit to eat fruit/vegetables daily  *(-2) fully disagree-(2) fully agree* | | | 2 (1-2) | | 2 (1-2) | | 2 (1-2) | | 2 (1-2) | |

Comparison within the educational groups of each country with Wilcoxon signed rank test. Rounded values are presented.

T_0_-T_1_: changes between pre and post-intervention period

*: significant within-group differences at .05

a: Negligible difference were found between the tow groups.
