# Supplementary material for: Inequalities in energy-balance related behaviours and family environmental determinants in European children: changes and sustainability within the EPHE evaluation study
Source: Int J Equity Health. 2016 Sep 29;15:160. doi: 10.1186/s12939-016-0438-1 (PMC5041563; doi:10.1186/s12939-016-0438-1)
Supplement: Additional file 2: — Within-group changes (T0-T1) in median values (q1-q3) in the determinants of fruit juices consumption. (DOCX 18 kb) [file 12939_2016_438_MOESM2_ESM.docx]

**Additional file 2**. Within-group changes (T_0_-T_1_) in median values (q_1_-q_3_) in the determinants of fruit juices consumption.

| **Fruit juices consumption** | | | | | | |
| --- | --- | --- | --- | --- | --- | --- |
| **Determinants**  **by country** | T_0_ | | | T_1_ | | |
| **Education level**  **Belgium** | | High | Low | | High | Low |
| Parental allowance  *never (0)-always (4)* | | 2 (1-3) | **3 (2-4)***** | | 2 (1-3) | **2 (1-3)** ******* |
| **Education level**  **France** | | High | Low | | High | Low |
| Nagging behaviour  *Never (0 )-yes, always (4)* | | 0 (0-0) | 0 (0-1) | | 0 (0-0) | 0 (0-1) |
| **Education level**  **Greece** | | High | Low | | High | Low |
| Negotiating  *never (0)-always (4)* | | 2 (1-3) | **3 (2-4)**** | | 2 (0-3) | **2 (1-3)**** |
| **Education level**  **Portugal** | | High | Low | | High | Low |
| Rewarding/comforting practice  *never (0)-always (4)* | | 0 (0-0) | **0 (0-1)*** | | 0 (0-0) | **0 (0-1)** |
| **Education level**  **Romania** | | High | Low | | High | Low |
| Paying attention/monitoring  *never (0)-always (4)* | | 3 (3-4) | 3 (2-4) | | 4 (3-4) | 3 (2-4) |
| Parental self- efficacy to retain rules  *never (0)-always (4)* | | 0 (0-1) | 1 (0-2) | | 0 (0-1) | 0 (0-2) |
| **Education level**  **The Netherlands** | | High | Low | | High | Low |
| Parental self- efficacy to retain rules  *never (0)-always (4)* | | 0 (0-0) | 0 (0-0) | | 0 (0-0) | 0 (0-1) |
| Rewarding/comforting practice  *never (0)-always (4)* | | 0 (0-0) | 0 (0-0) | | 0 (0-0) | 0 (0-0) |
| Nagging behaviour  *Never (0 )-yes, always (4)* | | 0 (0-0) | 0 (0-0) | | 0 (0-1) | 0 (0-2) |

Comparison within the educational groups of each country with Wilcoxon signed rank test. Rounded values are presented.

T_0_-T_1_: changes between pre and post-intervention period

*,**,***: significant within-group differences at .05, .01 and .001 respectively
