# Supplementary material for: Inequalities in energy-balance related behaviours and family environmental determinants in European children: changes and sustainability within the EPHE evaluation study
Source: Int J Equity Health. 2016 Sep 29;15:160. doi: 10.1186/s12939-016-0438-1 (PMC5041563; doi:10.1186/s12939-016-0438-1)
Supplement: Additional file 3: — Within-group changes (T0-T1) in median values (q1-q3) in the determinants of soft drinks consumption. (DOCX 18 kb) [file 12939_2016_438_MOESM3_ESM.docx]

**Additional file 3**. Within-group changes (T_0_-T_1_) in median values (q_1_-q_3_) in the determinants of soft drinks consumption.

| **Determinants**  **by country** | **Soft drinks consumption** | | | |
| --- | --- | --- | --- | --- |
|  | T_0_ | | T_1_ | |
| **Education level**  **Bulgaria** | High | Low | High | Low |
| Home availability  *never (0)-always (4)* | 1 (0-2) | 2 (0-2) | 1 (0-2) | 1 (0-3) |
| **Education level**  **France** | High | Low | High | Low |
| Nagging behaviour  *Never (0 )-yes, always (4)* | **0 (0-0)**** | 0 (0-0) | **0 (0-1)**** | 0 (0-0) |
| **Education level**  **Portugal** | High | Low | High | Low |
| Performing EBRB together with the child  *Never (0 )- always (4)* | 2 (1-2) | 2 (2-3) | 2 (1-3) | 2 (2-3) |
| Nagging behaviour  *Never (0 )-yes, always (4)* | 0 (0-1) | 0 (0-1) | 0 (0-1) | 0 (0-1) |
| Home availability  *never (0)-always (4)* | 1 (1-2) | **2 (1-2)*** | 1 (1-2) | **1 (1-2)*** |
| **Education level**  **Romania** | High | Low | High | Low |
| Parental allowance  *never (0)-always (4)* | 1 (0-2)  0 (0-1) | 1 (1-2)  1 (0-2) | 1 (1-1)  1 (0-1) | 2 (1-2)  1 (0-2) |
| Avoid negative modelling  *never (0)-always (4)* | 3 (2-4) | **2 (1-3)*** | 3 (1-4) | **2 (0-3)** |
| Performing EBRB together with the child  *Never (0 )-always (4)* | 2 (1-3) | 3 (2-3) | 2 (1-2) | 3 (2-4) |
| Home availability  *never (0)-always (4)* | 1 (0-2) | 1 (1-2) | 0 (0-1) | 1 (1-2) |

Comparison within the educational groups of each country with Wilcoxon signed rank test. Rounded values are presented.

T_0_-T_1_: changes between pre and post-intervention period

*,**: significant within-group differences at .05 and . 01respectively
