# Supplementary material for: Inequalities in energy-balance related behaviours and family environmental determinants in European children: changes and sustainability within the EPHE evaluation study
Source: Int J Equity Health. 2016 Sep 29;15:160. doi: 10.1186/s12939-016-0438-1 (PMC5041563; doi:10.1186/s12939-016-0438-1)
Supplement: Additional file 4: — Within-group changes (T0-T1) in median values (q1-q3) in the determinants of TV exposure. (DOCX 21 kb) [file 12939_2016_438_MOESM4_ESM.docx]

**Additional file 4**. Within-group changes (T_0_-T_1_) in median values (q_1_-q_3_) in the determinants of television (TV) exposure.

| **Determinants**  **by country** | **TV exposure** | | | |
| --- | --- | --- | --- | --- |
|  | **T_0_** | | **T_1_** | |
| **Education group**  **Belgium** | High | Low | High | Low |
| Paying attention/monitoring  *never (0)-always (4)* | 3 (2-4) | **3 (1-4)*** | 3 (2-4) | **3 (2-4)*** |
| Performing EBRB together with the child  *Never (0)- every day, more than once a day (7)* | 2 (2-3) | 3 (2-3) | 2 (2-3) | 3 (2-3) |
| TV in child’s bedroom  *yes* ***(%)*** | 14,0 | 31,0 | 8,3 | 29,0 |
| TV on during mealtime  *Every day (1)-never (6)* | 1 (1-5) | 1 (1-4) | 2 (1-5) | 1 (1-3) |
| **Education group**  **Bulgaria** |  |  |  |  |
| TV in child’s bedroom  *yes* ***(%)*** | 43,0 | 55,8 | 49,6 | 58,1 |
| **Education group**  **France** | High | Low | High | Low |
| Negotiating  *never (0)-always (4)* | **0 (0-3)*** | 2 (0-3) | **2 (0-3)*** | 1 (0-3) |
| Performing EBRB together with the child  *Never (0)- every day, more than once a day (7)* | 2 (2-3) | 3 (2-3) | 2 (2-3) | 3 (2-3) |
| **Education group**  **Greece** | High | Low | High | Low |
| TV on during meal time  *every day (1)-never (6)* | 5 (3-5) | 4 (2-5) | 4 (3-5) | 4 (3-5) |
| Parental self- efficacy to manage child’s exposure  *never (0)-always (4)* | 1 (0-1) | **1 (0-2)*** | 1 (0-1) | **1 (0-1)*** |
| **Education group**  **Portugal** | High | Low | High | Low |
| Rewarding/comforting practice  *never (0)-always (4)* | 1 (0-1) | 1(0-2) | 0 (0-1) | 1 (0-2) |
| Parental allowance  *never (0)-always (4)* | 2 (1-2) | **2 (2-3)*** | 2 (1-2) | **2 (1-2)*** |
| Nagging behaviour  *Never (0)-yes, always (4)* | 0 (0-1) | 1 (0-2) | 0 (0-1) | 1 (0-1) |
| TV on during meal time  *every day (1)-never (6)* | 1 (1-5) | 1 (1-2) | 1 (1-5) | 1 (1-3) |
| TV in child’s bedroom  *yes* ***(%)*** | 36,7 | 73,7 | 34,8 | 73,7 |
| **Education group**  **Romania** | High | Low | High | Low |
| Parental allowance  *never (0)-always (4)* | 3 (2-3) | 2 (2-3) | 2 (2-3) | 2 (2-3) |
| Negotiating  *never (0)-always (4)* | 2 (1-3) | 2 (0-2) | 2 (1-4) | 2 (0-3) |
| Performing EBRB together with the child  *Never (0)- every day, more than once a day (7)* | **2 (2-3)**** | 2 (2-3) | **2 (1-3)**** | 3 (2-3) |
| TV in child’s bedroom  *yes* ***(%)*** | 47,0 | 75,3 | 49,4 | 71,0 |
| **Education group**  **The Netherlands** | High | Low | High | Low |
| Paying attention/ monitoring  *never (0)-always (4)* | 3 (3-3) | 3 (2-3) | 3 (3-4) | 3 (3-4) |
| Parental allowance  *never (0)-always (4)* | 2 (2-3) | 2 (2-3) | 2 (2-3) | 3 (2-3) |
| Avoid negative modelling  *never (0)-always (4)* | 2 (1-3) | 2 (0-3) | 2 (1-3) | 2 (0-2) |
| TV in child’s bedroom  *yes* ***(%)*** | 4,4 | 26,3 | 6,5 | 15,7 |

Comparison within the educational groups of each country with Wilcoxon signed rank test. Rounded values are presented.

T_0_-T_1_: changes between pre and post-intervention period

*,**: significant within-group differences at .05 and .01 respectively
