# Supplementary material for: Inequalities in energy-balance related behaviours and family environmental determinants in European children: changes and sustainability within the EPHE evaluation study
Source: Int J Equity Health. 2016 Sep 29;15:160. doi: 10.1186/s12939-016-0438-1 (PMC5041563; doi:10.1186/s12939-016-0438-1)
Supplement: Additional file 5: — Within-group changes (T0-T1) in median values (q1-q3) in the determinants of PC exposure. . (DOCX 18 kb) [file 12939_2016_438_MOESM5_ESM.docx]

**Additional file 5.** Within-group changes (T_0_-T_1_) in median values (q_1_-q_3_) in the determinants of computer (PC) exposure.

| **Determinants**  **by country** | | **PC exposure** | | | | | | |
| --- | --- | --- | --- | --- | --- | --- | --- | --- |
|  |  | T_0_ | | T_1_ | | | |  |
| **Education level**  **Belgium** | | High | Low | High | | Low | |  |
| Performing EBRB together with the child  *Never (0)- every day, more than once a day (7)* | | 1 (0-2) | 2 (0-2) | 1 (0-2) | | 1 (1-3) | |  |
| Nagging behaviour  *Never (0)-yes, always (4)* | | 0 (0-0) | 0 (0-0) | 0 (0-0) | | 0 (0-0) | |  |
| **Education level**  **Bulgaria** | High | | Low | | High | | Low |  |
| Performing EBRB together with the child  *Never (0)- every day, more than once a day (7)* | 1 (0-2) | | 1 (0-3) | | 1 (0-2) | | 2 (1-2) |  |
| **Education level**  **France** | High | | Low | | High | | Low |  |
| Nagging behaviour  *Never (0)-yes, always (4)* | 0 (0-0) | | 0 (0-1) | | 0 (0-0) | | 0 (0-1) |  |
| **Education level**  **Romania** | High | | Low | | High | | Low |  |
| Negotiating  *never (0)-always (4)* | 3 (1-3) | | 2 (0-3) | | 3 (0-3) | | 2 (0-3) |  |
| **Education level**  **The Netherlands** | High | | Low | | High | | Low |  |
| Negotiating  *never (0)-always (4)* | 3 (3-4) | | 3 (2-4) | | 3 (2-4) | | 3 (3-4) |  |
| Avoid negative modelling  *never (0)-always (4)* | 2 (1-3) | | **2 (2-3)*** | | 2 (1-3) | | 2 **(0-2)*** |  |

Comparison within the educational groups of each country with Wilcoxon signed rank test. Rounded values are presented.

T_0_-T_1_: changes between pre and post-intervention period

* significant within group differences at .05
