# Supplementary material for: Inequalities in energy-balance related behaviours and family environmental determinants in European children: changes and sustainability within the EPHE evaluation study
Source: Int J Equity Health. 2016 Sep 29;15:160. doi: 10.1186/s12939-016-0438-1 (PMC5041563; doi:10.1186/s12939-016-0438-1)
Supplement: Additional file 6: — Within-group changes (T1 - T2) in median values (q1-q3) in determinants per behavior. (DOCX 20 kb) [file 12939_2016_438_MOESM6_ESM.docx]

**Additional file 6**. Within-group changes (T_1_ - T_2_) in median values (q_1_-q_3_) in determinants per behavior.

| **Determinant (s)**  **by country** | | **Fruit juices consumption** | | | | | | | | | | | | | | | | | | |  |
| --- | --- | --- | --- | --- | --- | --- | --- | --- | --- | --- | --- | --- | --- | --- | --- | --- | --- | --- | --- | --- | --- |
|  |  | T_1_ | | | | | | | | | | | | | T_2_ | | | | | |  |
| **Education level**  **Belgium** | | | | | | High | | | | Low | | | | | | | High | | | Low |  |
| Parental allowance  *never (0)-always (4)* | | | | | | 2 (1-3) | | | | 2 (1-3) | | | | | | | 2 (1-3) | | | 3 (1-3) |  |
| **Education level**  **Greece** | | | | | | High | | | | Low | | | | | | | High | | | Low |  |
| Negotiating  *never (0)-always (4)* | | | | | | **2 (0-2)*** | | | | **2 (1-3)*** | | | | | | | **2 (1-3) *** | | | **3 (2-4)*** |  |
| **Education level**  **Portugal** | | | | | | High | | | | Low | | | | | | | High | | | Low |  |
| Rewarding/comforting practice  *never (0)-always (4)* | | | | | | 0 (0-0) | | | | 0 (0-1) | | | | | | | 0 (0-0) | | | 0 (0-1) |  |
| **Determinant (s)**  **by country** | | | | | **Soft drinks consumption** | | | | | | | | | | | | | | | | |
|  |  |  |  |  | T_1_ | | | | | | | | T_2_ | | | | | | | | |
| **Education level**  **Portugal** | | | | | High | | | | Low | | | | High | | | | | | Low | | |
| Home availability  *never (0)-always (4)* | | | | | 1 (1-2) | | | | 1 (1-2) | | | | 1 (1-2) | | | | | | 1 (1-2) | | |
| **Determinant (s)**  **by country** | | | | **TV exposure** | | | | | | | | | | | | | | | | |  |
|  |  |  |  | T_1_ | | | | | | | | | | T_2_ | | | | | | |  |
| **Education group**  **Belgium** | | | | High | | | Low | | | | | | | High | | | | Low | | |  |
| Paying attention/monitoring  *never (0)-always (4)* | | | | 3 (2-4) | | | 3 (2-4) | | | | | | | 3 (3-4) | | | | 3 (2-4) | | |  |
| **Education group**  **Greece** | | | | High | | | Low | | | | | | |  | | | |  | | |  |
| Parental self- efficacy to manage child’s exposure  *never (0)-always (4)* | | | | **1 (0-1)*** | | | 1 (0-1) | | | | | | | **1 (0-2)*** | | | | 0 (0-1) | | |  |
| **Education group**  **Portugal** | | | | High | | | Low | | | | | | | High | | | | Low | | |  |
| Parental allowance  *never (0)-always (4)* | | | | **2 (1-2)** | | | 2 (1-2) | | | | | | | **1 (1-2)*** | | | | 2 (1-3) | | |  |
| **Determinants**  **by country** | | | **PC exposure** | | | | | | | | | | | | | | | | | |  |
|  |  |  | T_1_ | | | | | | | | | T_2_ | | | | | | | | |  |
| **Education level**  **The Netherlands** | High | | | | | | | Low | | | High | | | | | Low | | | | |  |
| Avoid negative modelling  *never (0)-always (4)* | 2 (1-3) | | | | | | | 2 (0-2) | | | 2 (0-2) | | | | | 2 (1-3) | | | | |  |

Comparison between the educational groups of each country with Wilcoxon signed rank test. Rounded values are presented.

T_1_-T_2_: changes between post-intervention and follow-up (a year after) period

*,**,***: significant within-group differences at .05, .01 and .001 respectively
