# Supplementary material for: Inequalities in energy-balance related behaviours and family environmental determinants in European children: changes and sustainability within the EPHE evaluation study
Source: Int J Equity Health. 2016 Sep 29;15:160. doi: 10.1186/s12939-016-0438-1 (PMC5041563; doi:10.1186/s12939-016-0438-1)
Supplement: Additional file 7: — Corrected critical p-values after adjustment for multiple testing (T1). (DOCX 34 kb) [file 12939_2016_438_MOESM7_ESM.docx]

**Additional file 7**. Corrected critical p-values after adjustment for multiple testing (T_1_).

| **Country** | **Belgium** | **Bulgaria** | **France** | **Greece** | **Portugal** | **Romania** | **Netherlands** | **TOTAL** |
| --- | --- | --- | --- | --- | --- | --- | --- | --- |
| **Differences within the high education group** | | | | | | | | |
| **Corrected overall critical**  **p-value**  **Energy balance**  **related-behaviour** | **0.002521** | **0.002532** | **0.000316** | **0.000316** | **0.000316** | **0.002215** | **0.000316** |  |
| Fruit consumption frequency (per week) | 0 | 0 | 0 | 0 | 0 | 0 | 0 |  |
| Salad or grated vegetables frequency (per week) | 1 | 0 | 0 | 0 | 0 | 0 | 0 |  |
| Raw vegetables frequency (per week) | 0 | 0 | 0 | 0 | 0 | 0 | 0 |  |
| Cooked Vegetables | 0 | 0 | 0 | 0 | 0 | 0 | 0 |  |
| Water frequency | 0 | 0 | 0 | 0 | 0 | 0 | 0 |  |
| Fruit juices frequency (per week) | 0 | 0 | 0 | 0 | 0 | 0 | 0 |  |
| Fruit juices' amount | 0 | 0 | 0 | 0 | 0 | 0 | 0 |  |
| Soft drinks frequency | 0 | 0 | 0 | 0 | 0 | 0 | 0 |  |
| Soft drinks amount | 0 | 0 | 0 | 0 | 0 | 0 | 0 |  |
| TV weekdays | 0 | 0 | 0 | 0 | 0 | 0 | 0 |  |
| TV weekend days | 0 | 0 | 0 | 0 | 0 | 0 | 0 |  |
| PC weekdays | 0 | 1 | 0 | 0 | 0 | 0 | 0 |  |
| PC weekend days | 0 | 0 | 0 | 0 | 0 | 0 | 0 |  |
| Total screen time | 0 | 0 | 0 | 0 | 0 | 0 | 0 |  |
| Sleep hours-Week days | 0 | 0 | 0 | 0 | 0 | 0 | 0 |  |
| Sleep hours-Weekend days | 0 | 0 | 0 | 0 | 0 | 0 | 0 |  |
| **Determinants of fruit consumption** | | | | | | | | |
| Parental knowledge on recommendations | 0 | 0 | 0 | 0 | 0 | 0 | 0 |  |
| Active encouragement | 0 | 1 | 0 | 0 | 0 | 0 | 0 |  |
| Performing EBRB together with the child | 0 | 0 | 0 | 0 | 0 | 0 | 0 |  |
| Habit to eat fruit daily | 0 | 0 | 0 | 0 | 0 | 0 | 0 |  |
| Parental demand | 0 | 0 | 0 | 0 | 0 | 0 | 0 |  |
| Parental allowance | 0 | 0 | 0 | 0 | 0 | 0 | 0 |  |
| Home availability | 0 | 0 | 0 | 0 | 0 | 0 | 0 |  |
| Parental facilitation | 0 | 0 | 0 | 0 | 0 | 0 | 0 |  |
| **Determinants of vegetable consumption** | | | | | | | | |
| Parental knowledge on recommendations | 0 | 0 | 0 | 0 | 0 | 0 | 0 |  |
| Active encouragement | 0 | 1 | 0 | 0 | 0 | 0 | 0 |  |
| Performing EBRB together with the child | 0 | 1 | 0 | 0 | 0 | 0 | 0 |  |
| Habit to eat vegetables daily | 0 | 0 | 0 | 0 | 0 | 0 | 0 |  |
| Parental demand | 0 | 0 | 0 | 0 | 0 | 0 | 0 |  |
| Parental allowance | 0 | 0 | 0 | 0 | 0 | 0 | 0 |  |
| Home availability | 0 | 1 | 0 | 0 | 0 | 0 | 0 |  |
| Parental facilitation | 1 | 0 | 0 | 0 | 0 | 0 | 0 |  |
| **Determinants of fruit juices consumption** | | | | | | | | |
| Home availability | 0 | 0 | 0 | 0 | 0 | 0 | 0 |  |
| Paying attention/monitoring | 0 | 0 | 0 | 0 | 0 | 0 | 0 |  |
| Parental allowance 1 | 0 | 0 | 0 | 0 | 0 | 0 | 0 |  |
| Parental allowance 2 | 0 | 0 | 0 | 0 | 0 | 0 | 0 |  |
| Negotiating | 0 | 0 | 0 | 0 | 0 | 0 | 0 |  |
| Communicating the health belief 1 | 0 | 0 | 0 | 0 | 0 | 0 | 0 |  |
| Communicating the health belief 2 | 0 | 0 | 0 | 0 | 0 | 0 | 0 |  |
| Avoid negative modelling | 0 | 0 | 0 | 0 | 0 | 0 | 0 |  |
| Nagging behaviour | 0 | 0 | 0 | 0 | 0 | 0 | 0 |  |
| Parental self- efficacy to retain rules | 0 | 0 | 0 | 0 | 0 | 0 | 0 |  |
| Rewarding/comforting practice | 0 | 0 | 0 | 0 | 0 | 0 | 0 |  |
| Conducting energy-balance related behaviour together with the child | 0 | 0 | 0 | 0 | 0 | 0 | 0 |  |
| **Determinants of soft drinks consumption** | | | | | | | | |
| Home availability | 0 | 0 | 0 | 0 | 0 | 0 | 0 |  |
| Paying attention/monitoring | 0 | 0 | 0 | 0 | 0 | 0 | 0 |  |
| Parental allowance 1 | 0 | 0 | 0 | 0 | 0 | 0 | 0 |  |
| Parental allowance 2 | 0 | 0 | 0 | 0 | 0 | 0 | 0 |  |
| Communicating health belief 1 | 0 | 0 | 0 | 0 | 0 | 0 | 0 |  |
| Communicating health belief 2 | 0 | 0 | 0 | 0 | 0 | 0 | 0 |  |
| Avoid negative modelling | 0 | 0 | 0 | 0 | 0 | 0 | 0 |  |
| Nagging behaviour | 0 | 0 | 0 | 0 | 0 | 0 | 0 |  |
| Parental self- efficacy to retain rules | 0 | 0 | 0 | 0 | 0 | 0 | 0 |  |
| Rewarding/comforting practice | 0 | 0 | 0 | 0 | 0 | 0 | 0 |  |
| Conducting energy-balance related behaviour together with the child | 0 | 0 | 0 | 0 | 0 | 0 | 0 |  |
| **Determinants of television exposure** | | | | | | | | |
| TV in child’s bedroom | 0 | 0 | 0 | 0 | 0 | 0 | 0 |  |
| TV on during meal time | 0 | 0 | 0 | 0 | 0 | 0 | 0 |  |
| Paying attention/monitoring | 0 | 0 | 0 | 0 | 0 | 0 | 0 |  |
| Parental allowance 1 | 0 | 0 | 0 | 0 | 1 | 0 | 0 |  |
| Parental allowance 2 | 0 | 0 | 0 | 0 | 0 | 0 | 0 |  |
| Negotiating | 0 | 0 | 0 | 0 | 0 | 0 | 0 |  |
| Avoid negative modelling | 0 | 0 | 0 | 0 | 1 | 0 | 0 |  |
| Nagging behaviour | 0 | 0 | 0 | 0 | 0 | 0 | 0 |  |
| Parental self- efficacy to retain rules | 0 | 0 | 0 | 0 | 0 | 0 | 0 |  |
| Rewarding/comforting practice | 0 | 0 | 0 | 0 | 0 | 0 | 0 |  |
| Performing energy-balance related behaviour together with the child | 0 | 0 | 0 | 0 | 0 | 0 | 0 |  |
| Communicating health belief 1 (TV and PC exposure) | 0 | 0 | 0 | 0 | 0 | 0 | 0 |  |
| Communicating health belief 2 (TV and PC exposure) | 0 | 0 | 0 | 0 | 0 | 0 | 0 |  |
| **Determinants of computer exposure** | | | | | | | | |
| Paying attention/monitoring | 0 | 0 | 0 | 0 | 0 | 0 | 0 |  |
| Parental allowance 1 | 0 | 0 | 0 | 0 | 0 | 0 | 0 |  |
| Parental allowance 2 | 0 | 0 | 0 | 0 | 0 | 0 | 0 |  |
| Negotiating | 0 | 0 | 0 | 0 | 0 | 0 | 0 |  |
| Avoid negative modelling | 0 | 0 | 0 | 0 | 0 | 0 | 0 |  |
| Nagging behaviour | 0 | 0 | 0 | 0 | 1 | 0 | 0 |  |
| Parental self- efficacy to retain rules | 0 | 0 | 0 | 0 | 0 | 0 | 0 |  |
| Rewarding/comforting practice | 0 | 0 | 0 | 0 | 0 | 0 | 0 |  |
| Performing energy-balance related behaviour together with the child | 0 | 0 | 0 | 0 | 0 | 0 | 0 |  |
| **Country** | **Belgium** | **Bulgaria** | **France** | **Greece** | **Portugal** | **Romania** | **Netherlands** | **TOTAL** |
| **Differences within the low education group** | | | | | | | | |
| **Corrected overall critical**  **p-value**  **Energy balance**  **related-behaviour** | **0.002521** | **0.002532** | **0.000316** | **0.000316** | **0.000316** | **0.002215** | **0.000316** |  |
| Fruit consumption frequency (per week) | 0 | 0 | 0 | 0 | 0 | 0 | 0 |  |
| Salad or grated vegetables frequency (per week) | 0 | 0 | 0 | 0 | 0 | 0 | 0 |  |
| Raw vegetables frequency (per week) | 0 | 0 | 0 | 0 | 0 | 0 | 0 |  |
| Cooked Vegetables | 0 | 0 | 0 | 0 | 0 | 0 | 0 |  |
| Water frequency | 0 | 0 | 0 | 0 | 0 | 0 | 0 |  |
| Fruit juices frequency (per week) | 0 | 0 | 0 | 0 | 0 | 0 | 0 |  |
| Fruit juices' amount | 0 | 0 | 0 | 0 | 0 | 0 | 0 |  |
| Soft drinks frequency | 0 | 0 | 0 | 0 | 0 | 0 | 0 |  |
| Soft drinks amount | 0 | 0 | 0 | 0 | 0 | 0 | 0 |  |
| TV weekdays | 1 | 0 | 0 | 0 | 0 | 0 | 0 |  |
| TV weekend days | 0 | 0 | 0 | 0 | 0 | 0 | 0 |  |
| PC weekdays | 0 | 0 | 0 | 0 | 0 | 1 | 0 |  |
| PC weekend days | 0 | 0 | 0 | 1 | 0 | 1 | 0 |  |
| Total screen time | 0 | 0 | 0 | 0 | 0 | 0 | 0 |  |
| Sleep hours-Week days | 0 | 0 | 0 | 0 | 0 | 0 | 0 |  |
| Sleep hours-Weekend days | 0 | 0 | 0 | 0 | 0 | 0 | 0 |  |
| **Determinants of fruit consumption** | | | | | | | | |
| Parental knowledge on recommendations | 0 | 0 | 0 | 0 | 0 | 0 | 0 |  |
| Active encouragement | 0 | 1 | 0 | 0 | 0 | 0 | 0 |  |
| Performing EBRB together with the child | 0 | 0 | 0 | 0 | 0 | 0 | 0 |  |
| Habit to eat fruit daily | 0 | 0 | 0 | 0 | 0 | 0 | 0 |  |
| Parental demand | 0 | 0 | 0 | 0 | 0 | 0 | 0 |  |
| Parental allowance | 0 | 0 | 0 | 0 | 0 | 0 | 0 |  |
| Home availability | 0 | 0 | 0 | 0 | 0 | 0 | 0 |  |
| Parental facilitation | 0 | 0 | 0 | 0 | 0 | 0 | 0 |  |
| **Determinants of vegetable consumption** | | | | | | | | |
| Parental knowledge on recommendations | 0 | 0 | 0 | 0 | 0 | 0 | 0 |  |
| Active encouragement | 0 | 1 | 0 | 0 | 0 | 0 | 0 |  |
| Performing EBRB together with the child | 0 | 1 | 0 | 0 | 0 | 0 | 0 |  |
| Habit to eat vegetables daily | 0 | 0 | 0 | 0 | 0 | 0 | 0 |  |
| Parental demand | 0 | 0 | 0 | 0 | 0 | 0 | 0 |  |
| Parental allowance | 0 | 0 | 0 | 0 | 0 | 0 | 0 |  |
| Home availability | 0 | 0 | 0 | 0 | 0 | 0 | 0 |  |
| Parental facilitation | 0 | 0 | 0 | 0 | 0 | 0 | 0 |  |
| **Determinants of fruit juices consumption** | | | | | | | | |
| Home availability | 1 | 0 | 0 | 0 | 0 | 0 | 0 |  |
| Paying attention/monitoring | 0 | 0 | 0 | 0 | 0 | 0 | 0 |  |
| Parental allowance 1 | 1 | 0 | 0 | 0 | 0 | 0 | 0 |  |
| Parental allowance 2 | 1 | 0 | 0 | 0 | 0 | 0 | 0 |  |
| Negotiating | 0 | 0 | 0 | 0 | 0 | 0 | 0 |  |
| Communicating the health belief 1 | 0 | 0 | 0 | 0 | 0 | 0 | 0 |  |
| Communicating the health belief 2 | 0 | 0 | 0 | 0 | 0 | 0 | 0 |  |
| Avoid negative modelling | 0 | 0 | 0 | 0 | 0 | 0 | 0 |  |
| Nagging behaviour | 0 | 0 | 0 | 0 | 0 | 0 | 0 |  |
| Parental self- efficacy to retain rules | 0 | 0 | 0 | 0 | 0 | 0 | 0 |  |
| Rewarding/comforting practice | 0 | 0 | 0 | 0 | 0 | 0 | 0 |  |
| Conducting energy-balance related behaviour together with the child | 0 | 0 | 0 | 0 | 0 | 0 | 0 |  |
| **Determinants of soft drinks consumption** | | | | | | | | |
| Home availability | 0 | 0 | 0 | 0 | 0 | 0 | 0 |  |
| Paying attention/monitoring | 0 | 0 | 0 | 0 | 0 | 0 | 0 |  |
| Parental allowance 1 | 0 | 0 | 0 | 0 | 0 | 0 | 0 |  |
| Parental allowance 2 | 0 | 0 | 0 | 0 | 0 | 0 | 0 |  |
| Communicating health belief 1 | 0 | 0 | 0 | 0 | 0 | 0 | 0 |  |
| Communicating health belief 2 | 0 | 0 | 0 | 0 | 0 | 0 | 0 |  |
| Avoid negative modelling | 0 | 0 | 0 | 0 | 0 | 0 | 0 |  |
| Nagging behaviour | 0 | 0 | 0 | 0 | 0 | 0 | 0 |  |
| Parental self- efficacy to retain rules | 0 | 0 | 0 | 0 | 0 | 0 | 0 |  |
| Rewarding/comforting practice | 0 | 0 | 0 | 0 | 0 | 0 | 0 |  |
| Conducting energy-balance related behaviour together with the child | 0 | 0 | 0 | 0 | 0 | 0 | 0 |  |
| **Determinants of television exposure** | | | | | | | | |
| TV in child’s bedroom | 0 | 0 | 0 | 0 | 0 | 0 | 0 |  |
| TV on during meal time | 0 | 0 | 0 | 0 | 0 | 0 | 0 |  |
| Paying attention/monitoring | 0 | 0 | 0 | 0 | 0 | 0 | 0 |  |
| Parental allowance 1 | 0 | 0 | 0 | 0 | 0 | 0 | 0 |  |
| Parental allowance 2 | 0 | 0 | 0 | 0 | 0 | 0 | 0 |  |
| Negotiating | 0 | 0 | 0 | 0 | 0 | 0 | 0 |  |
| Avoid negative modelling | 0 | 0 | 0 | 0 | 0 | 1 | 0 |  |
| Nagging behaviour | 0 | 0 | 0 | 0 | 0 | 0 | 0 |  |
| Parental self- efficacy to retain rules | 0 | 0 | 0 | 0 | 0 | 0 | 0 |  |
| Rewarding/comforting practice | 0 | 0 | 0 | 0 | 0 | 0 | 0 |  |
| Performing energy-balance related behaviour together with the child | 0 | 0 | 0 | 0 | 0 | 0 | 0 |  |
| Communicating health belief 1 (TV and PC exposure) | 0 | 0 | 0 | 0 | 0 | 0 | 0 |  |
| Communicating health belief 2 (TV and PC exposure) | 0 | 0 | 0 | 0 | 0 | 0 | 0 |  |
| **Determinants of computer exposure** | | | | | | | | |
| Paying attention/monitoring | 0 | 0 | 0 | 0 | 0 | 0 | 0 |  |
| Parental allowance 1 | 0 | 0 | 0 | 0 | 0 | 0 | 0 |  |
| Parental allowance 2 | 0 | 0 | 0 | 0 | 0 | 0 | 0 |  |
| Negotiating | 0 | 0 | 0 | 0 | 0 | 0 | 0 |  |
| Avoid negative modelling | 0 | 0 | 0 | 0 | 0 | 0 | 0 |  |
| Nagging behaviour | 0 | 0 | 0 | 0 | 0 | 1 | 0 |  |
| Parental self- efficacy to retain rules | 0 | 0 | 0 | 0 | 0 | 0 | 0 |  |
| Rewarding/comforting practice | 0 | 0 | 0 | 0 | 0 | 0 | 0 |  |
| Performing energy-balance related behaviour together with the child | 0 | 0 | 0 | 0 | 0 | 0 | 0 |  |

Multiple testing adjustment by the Benjamini and Hocheberg method [44].

**0=**the adjusted p-value is higher than the corrected critical p-value. **1=** the adjusted p-value is lower than the corrected critical p-value.

T_1_: Post-intervention follow-up, after one school year intervention period.
